# The effects of Bi_2_O_3_ on the selective catalytic reduction of NO by propylene over Co_3_O_4_ nanoplates

**DOI:** 10.1039/c9ra03956b

**Published:** 2019-10-10

**Authors:** Dezhong Yu, Xin Zhong, Dong Liu, Ying Liang

**Affiliations:** School of Chemistry and Environmental Engineering, Wuhan Institute of Technology Wuhan 430205 China witchem2018@163.com; School of Chemical Engineering, Hubei University of Arts and Science Xiangyang 441053 China

## Abstract

Bi_2_O_3_/Co_3_O_4_ catalysts prepared by the impregnation method were investigated for the selective catalytic reduction of NO by C_3_H_6_ (C_3_H_6_-SCR) in the presence of O_2_. Their physicochemical properties were analyzed with SEM, XRD, H_2_-TPR, XPS, PL and IR measurements. It was found that the deposition of Bi_2_O_3_ on Co_3_O_4_ nanoplates enhanced the catalytic activity, especially at low reaction temperature. The SO_2_ tolerance of Co_3_O_4_ in C_3_H_6_-SCR activity was also improved with the addition of Bi_2_O_3_. Among all catalysts tested, 10.0 wt% Bi_2_O_3_/Co_3_O_4_ achieved a 90% NO conversion at 200 °C with the total flow rate of 200 mL min^−1^ (GHSV 30 000 h^−1^). No loss in its C_3_H_6_-SCR activity was observed at different temperatures after the addition of 100 ppm of SO_2_ to the reaction mixture. These enhanced catalytic behaviors may be associated with the improved oxidizing characteristics of 10.0 wt% Bi_2_O_3_/Co_3_O_2_. XRD results showed that Bi_2_O_3_ entered the lattice of Co_3_O_4_, resulting in the formation of lattice distortion and structural defects. H_2_-TPR results showed that the reduction of Co_3_O_4_ was promoted and the diffusion of oxygen was accelerated with the addition of Bi_2_O_3_. XPS measurements implied that more Co^3+^ formed on the 10.0% Bi_2_O_3_/Co_3_O_2_ catalysts. The improved oxidizing characteristics of the catalyst with the addition of Bi_2_O_3_ due to the synergistic effect of the nanostructure hybrid, thus enhanced the C_3_H_6_-SCR reaction and hindered the oxidization of SO_2_. Therefore, the 10.0% Bi_2_O_3_/Co_3_O_4_ catalyst exhibited the highest NO conversion and strongest SO_2_ tolerance ability.

## Abstract 2

Bi_2_O_3_/Co_3_O_4_ catalysts prepared by the impregnation method were investigated for the selective catalytic reduction of NO by C_3_H_6_ (C_3_H_6_-SCR) in the presence of O_2_.

## Graphical abstract

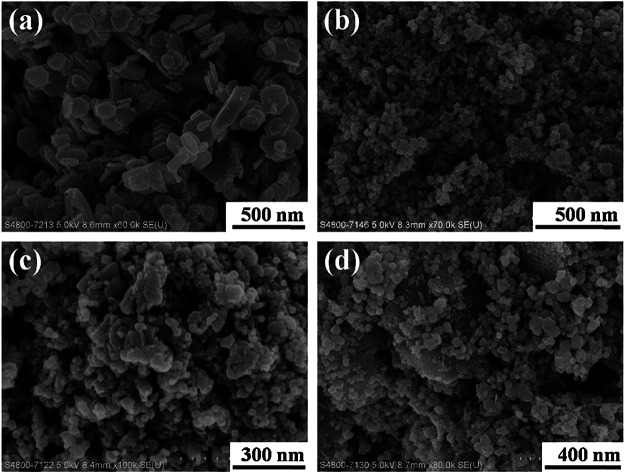

## 1 Introduction

Lean burn engines, which are generally used in gasoline and diesel powered vehicles, are more fuel-efficient than the stoichiometric gasoline engines.^1^ They also effectively reduce unburned hydrocarbons, CO_2_ and CO in exhausts.^2^ However, lean burn engines operate with a large excess of air, leading to a significant concentration of oxygen in the exhausts, where the noble-metal three-way catalysts cannot work well to reduce nitrogen oxides (NO_*x*_).^3^ A large amount of NO_*x*_ produced by lean burn engines leads to serious air pollution and public health problems.

In order to control NO_*x*_ emission under the lean burn conditions, selective catalytic reduction of NO by hydrocarbons (*e.g.* propylene) has been undertaken and reported in the literatures as one potential application (HC-SCR). Many classes of catalysts, including supported noble metals (*e.g.* Pt,^4,5^ Au^6,7^), metal oxides (*e.g.* Ag_2_O,^8,9^ CuO,^10,11^ SnO_2_,^12–14^ CoO_*x*_ (ref. 15–18)) and zeolite types (ZSM-5,^19^ MCM-41 (ref. 20)) have been investigated. In general, the noble metals are active and stable even at lower temperature, but the formation of N_2_O is undesirable by using such precious metals, particularly, platinum-based catalysts. The zeolite-based catalysts were low thermal stability. Among metal oxides catalysts, cobalt oxides (*e.g.* Co_3_O_4_) are considered as one promising catalyst for HC-SCR due to its high catalytic activity.^21^ When combined with other oxides, such as CeO_2_,^16^ ZrO_2_,^15^ Al_2_O_3_,^18^ sulphated ZrO_2_,^22^ the catalytic performances of the cobalt oxide catalyst could be improved as reported. These results implied that the chemical environment around cobalt oxide plays a crucial role in controlling the overall activity of cobalt containing catalysts in SCR reactions.

Bi_2_O_3_, a common oxide semiconductor, is widely used in the fields of chemical engineering and electronics such as NO detection^23^ and the oxidation or ammoxidation of propylene.^24,25^ In the oxidation/ammoxidation of propene over bismuth/molybdate catalyst, bismuth was thought to involve the rate-determining hydrogen abstraction from propylene,^26^ exhibiting its mild oxidizing characteristics. This property might be also beneficial for the partial oxidation of propylene in C_3_H_6_-SCR for NO reduction. Therefore, it is of considerable interest to explore the application of Bi_2_O_3_ in the reduction of NO with propylene.

In the present study, Co_3_O_4_ nanoplates and Bi_2_O_3_/Co_3_O_4_ were prepared with the solvothermal and impregnation method respectively. Their catalytic performances in the NO reduction by C_3_H_6_ in the presence of O_2_ were investigated. The catalysts were characterized with X-ray diffraction (XRD), temperature programmed reduction with hydrogen (H_2_-TPR), and X-ray photoelectron spectra (XPS). The effects of Bi_2_O_3_ on the selective catalytic reduction of NO by propylene over Co_3_O_4_ nanoplates were expected to be elucidated.

## 2 Experimental

### 2.1 Preparation of Co_3_O_4_ and Bi_2_O_3_/Co_3_O_4_

The Co_3_O_4_ support was synthesized *via* the solvent-thermal method, 50 mmol CoCl_2_ solution (200 mmol L^−1^, Sinopharm Chemical Reagent Co. China) and 25 mL of NaOH (2 mol L^−1^, Sinopharm Chemical Reagent Co. China) were added to a round-bottom flask, ultrasonicated for about 20 minutes to obtain a light brown uniform suspension. And then the suspension was transferred into a stainless steel autoclave with Teflon liner. The autoclave was sealed and maintained at 120 °C for 12 h. The obtained product was collected after washing with deionized water for several times, finally calcined at 500 °C for 5 h in air (S1).

The Bi_2_O_3_/Co_3_O_4_ catalysts with different Bi_2_O_3_ contents were synthesized by impregnation method as followed: 0.0155 g Bi(NO_3_)_3_·5H_2_O and 20 mL 3% NH_3_·H_2_O were added into a round-bottom flask, ultrasonicated for about 20 minutes to obtain a white uniform suspension. After that, the Co_3_O_4_ support (S1) were added in the suspension, ultrasonicated for about 15 minutes to make it homogeneously distributed in the suspension. The suspension was then dried at 80 °C with continuous stirring for 1 h, further heated at 120 °C for 12 h followed by calcination at 500 °C in air for 4 h, yielding the 5.0 wt% Bi_2_O_3_/Co_3_O_4_ catalyst (S2). 10.0 wt% Bi_2_O_3_/Co_3_O_4_ (S3) and 15.0 wt% Bi_2_O_3_/Co_3_O_4_ (S4) were prepared with 0.0310 g and 0.0465 g Bi(NO_3_)_3_·5H_2_O respectively. In addition, the physical mixture of 10% Bi_2_O_3_ nanoparticles and Co_3_O_4_ support was also prepared and labeled as S5. As references, classical catalysts 4% Ag/Al_2_O_3_ and 2% Pt/Al_2_O_3_ were prepared to compare the catalytic performance of the Bi_2_O_3_/Co_3_O_4_ catalysts.

### 2.2 Catalytic activity tests

C_3_H_6_-SCR over the catalysts was carried out at atmospheric pressure in a fixed-bed quartz reactor (diameter = 10 mm). 0.1 g catalyst was used in each run with a reaction mixture composed of 200 ppm NO, 200 ppm C_3_H_6_, 100 ppm SO_2_ (when needed) and 10 vol% O_2_ in balance gas N_2_. The total flow rate was 200 mL min^−1^, corresponding to a GHSV 30 000 h^−1^. Reaction temperature ranges from 100 to 500 °C. The concentration of NO was continuously measured by a NO analyzer (Thermo Environmental Instruments Inc., model 42c), which monitors NO, NO_2_, and NO_*x*_ (NO_*x*_ represents NO + NO_2_). The removal efficiency of NO was calculated as NO removal (%) = (1 − *C*/*C*_0_) × 100%, where *C* and *C*_0_ are concentrations of NO in the outlet and inlet, respectively.

### 2.3 Catalysts characterization

Scanning electron microscopy (SEM) images were taken on a Hitachi S4800 scanning electron microscope operating at 5.0 kV. X-ray powder diffraction (XRD) was carried out on Brukeraxs D8 Discover (Cu Kα = 1.5406 Å). The scanning rate is 1° min^−1^ in the 2*θ* range from 20 to 80 degree. The reducibility of Bi_2_O_3_/Co_3_O_4_ catalysts was estimated by temperature programmed reduction with hydrogen analysis (H_2_-TPR). The experiments were carried out with a Micromeritics 2910 apparatus using H_2_/Ar (3/97, v/v) gas with a total flow rate of 15 mL min^−1^. In each run, 0.030 g of the catalyst was previously activated at 500 °C for 30 min under air, and then cooled to RT. TPR started with the introduction of the mixture of H_2_ and Ar. The catalyst was heated from room temperature (RT) to 1000 °C (10 °C min^−1^). H_2_ consumption was continuously monitored with the thermal conductivity detector. X-ray photoelectron spectra (XPS) of the catalysts were measured in a VG Multilab 2000 spectrometer by using Al Kα (1486.6 eV) radiation as the X-ray source. Photoluminescence (PL) measurement was carried out on a Shimadzu RF-5301 PC fluorescence spectrophotometer. Raman spectra were recorded using a Horiba Jobin-Yvon Lab Ram HR800 Raman microspectrometer, with an excitation laser at 514 nm.

## 3 Results

### 3.1 Scanning electron microscope (SEM) observation and XRD analysis

**Fig. 1:**
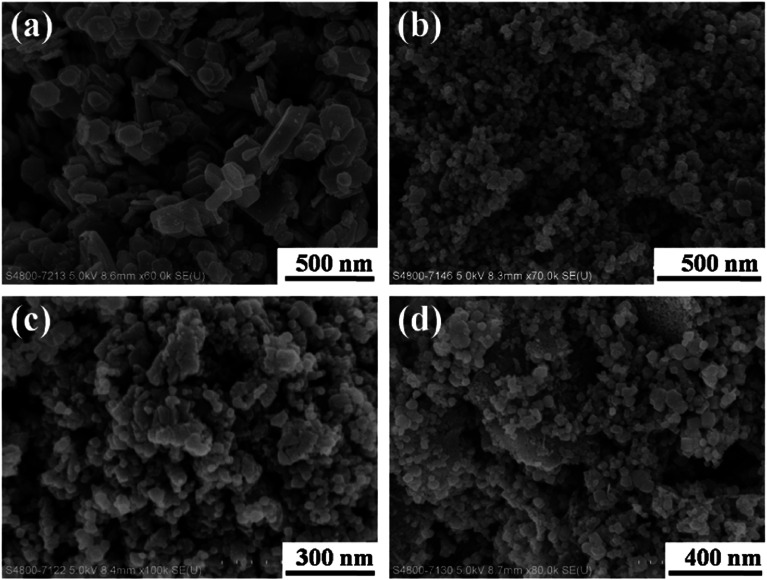
SEM images of Co_3_O_4_ (a), 5.0% Bi_2_O_3_/Co_3_O_4_ (b), 10.0% Bi_2_O_3_/Co_3_O_4_ (c) and 15.0% Bi_2_O_3_/Co_3_O_4_ (d).

Co_3_O_4_ nanoplates with different dimensions were observed on the support Co_3_O_4_ (S1), shown in Fig. 1(a). Fig. 1(b–d) presented Bi_2_O_3_/Co_3_O_4_ catalysts with different Bi_2_O_3_ loading amounts. It was shown that Bi_2_O_3_ nanoparticles with dimension *ca.* 20 nm were supported on Co_3_O_4_. And the crystal size of Bi_2_O_3_ slightly increased with increasing Bi_2_O_3_ loading amount from 5% to 15%.

**Fig. 2:**
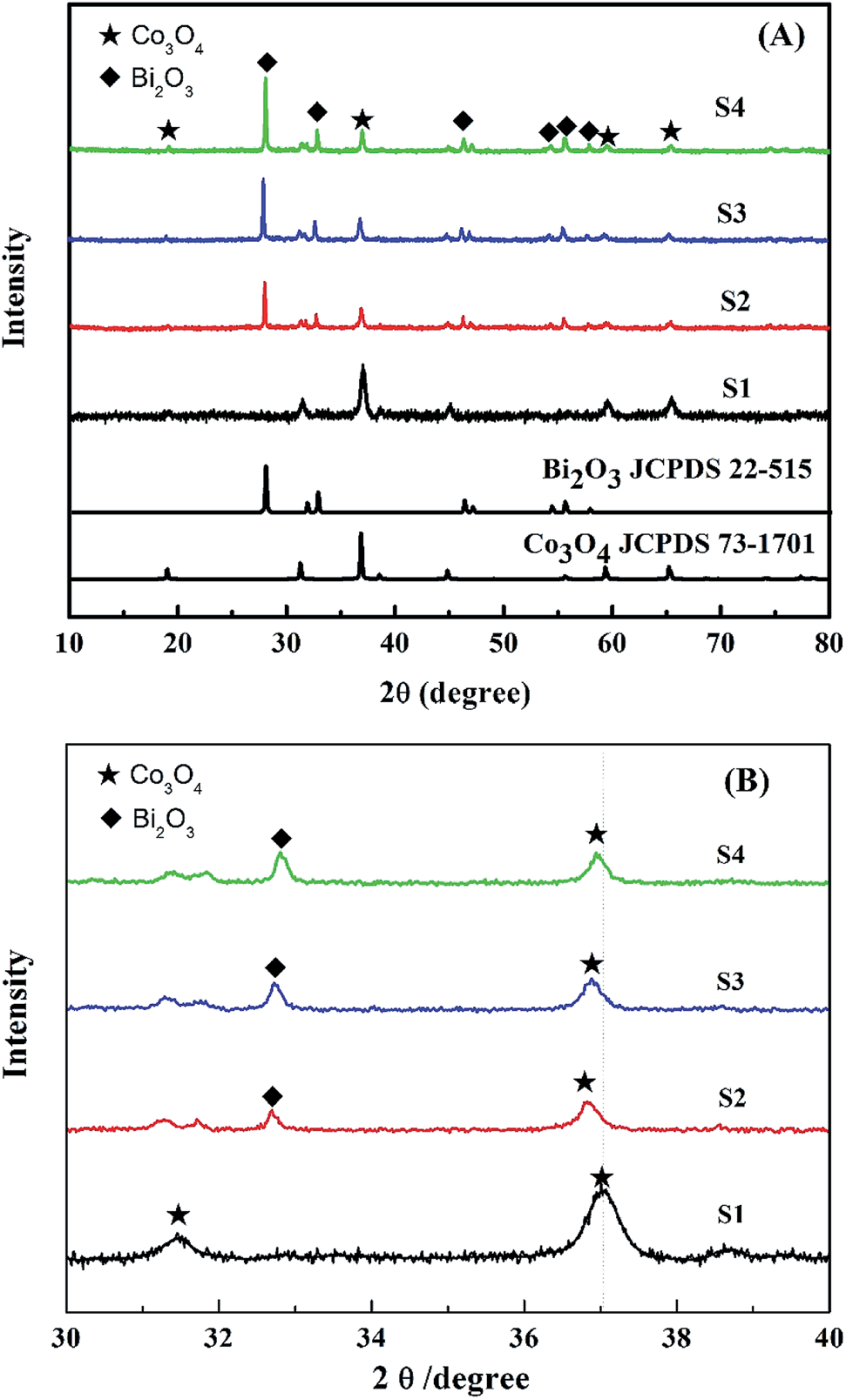
XRD patterns of Co_3_O_4_ (S1), 5.0% Bi_2_O_3_/Co_3_O_4_ (S2), 10.0% Bi_2_O_3_/Co_3_O_4_ (S3) and 15.0% Bi_2_O_3_/Co_3_O_4_ (S4) in the wide (A) and narrow (B) ranges.

Fig. 2(A) shows the XRD patterns of the synthesized Co_3_O_4_ and Bi_2_O_3_/Co_3_O_4_ catalysts. It can be seen that the Co_3_O_4_ support possessed the characteristic peaks of Co_3_O_4_ (JCPDS 73-1701, *a* = 5.45 Å). Besides the characteristic peaks of Co_3_O_4_, the diffraction peaks due to Bi_2_O_3_ (JCPDS 22-515, *a* = 10.94 Å and *c* = 11.28 Å) were also observed on Bi_2_O_3_/Co_3_O_4_ catalysts which became sharper with the increase of Bi_2_O_3_ loading amount. It was suggested that the crystal size of Bi_2_O_3_ was bigger on the catalyst with higher loading amount of Bi_2_O_3_, which was consistent with the SEM observations. Moreover, the diffraction peaks of Co_3_O_4_ over Bi_2_O_3_/Co_3_O_4_ catalysts behaved a slight shift towards lower degree in comparison with that of Co_3_O_4_, shown in Fig. 2(B). It indicated that the Bi^3+^ inserted the lattice of Co_3_O_4_ in the preparation process, and changed the lattice parameter of Co_3_O_4_ due to the different radius of Bi and Co atoms. At the same time, the characteristic peak of Co_3_O_4_ became weak obviously after the deposition of Bi_2_O_3_, which revealed the reduction of crystal size. It was implied that the insertion of Bi_2_O_3_ induced the structure defect of Co_3_O_4_ and suppressed the growth of crystal.

### 3.2 Temperature-programmed reduction by hydrogen (H_2_-TPR)

**Fig. 3:**
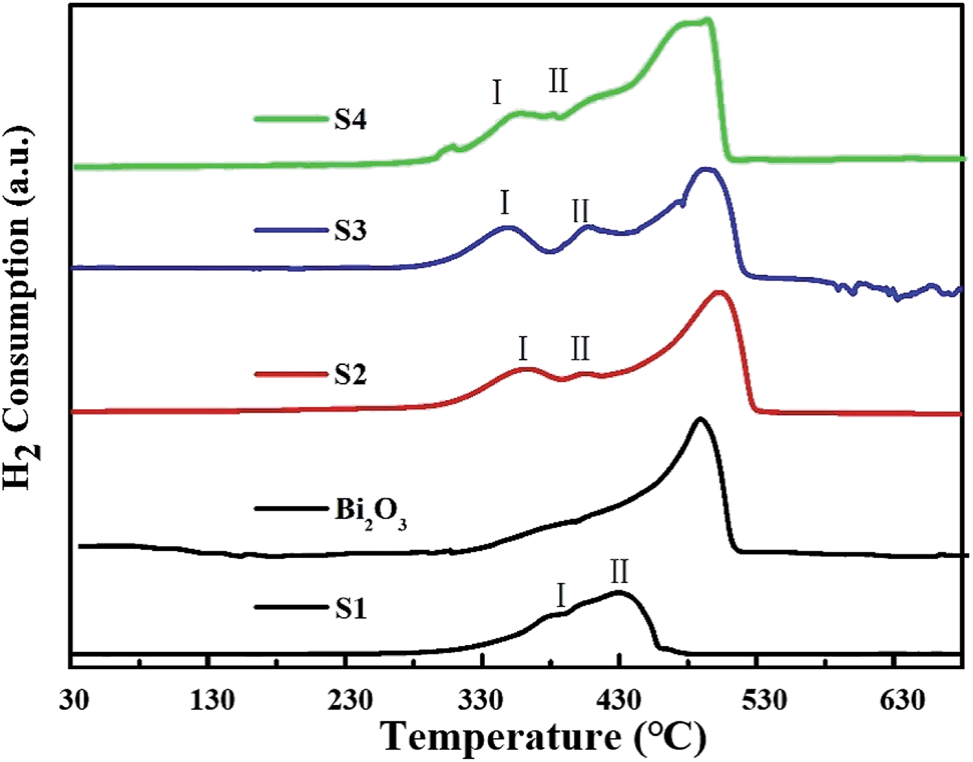
H_2_-TPR profiles of pure Co_3_O_4_ (S1), Bi_2_O_3_, 5.0% Bi_2_O_3_/Co_3_O_4_ (S2), 10.0% Bi_2_O_3_/Co_3_O_4_ (S3) and 15.0% Bi_2_O_3_/Co_3_O_4_ (S4).

The H_2_-TPR profiles of Co_3_O_4_, Bi_2_O_3_ and Bi_2_O_3_/Co_3_O_4_ catalysts were shown in Fig. 3. For the reduction of Bi_2_O_3_, a sharp peak was observed at *ca.* 490 °C, implying the reduction of Bi^3+^ in a narrow temperature range. A broad reduction peak from 330 to 460 °C with a large shoulder at the lower reduction temperature (*ca.* 380 °C) appeared on Co_3_O_4_ support (S1). Many researchers reported that the reduction of Co_3_O_4_ was a two-step reduction process involving the intermediate reduction of CoO.^16,21,27^ Two main clear reduction peaks respectively located around 186 °C and 310–480 °C were shown in the TPR spectra. The low temperature TPR peak was associated with the reduction of Co^3+^ to Co^2+^, and the peak at high temperature was the subsequent reduction of CoO to metallic cobalt. In the TPR spectrum of Co_3_O_4_ synthesized in the present work, there are no obvious two peaks probably due to an abroad particle size distribution as shown in SEM observation. The large shoulder at 376 °C (peak I) in Fig. 3 should be attributed to the reduction of Co^3+^ to Co^2+^, and main reduction peak (peak II) is ascribed to the reduction of CoO to metallic cobalt.

The reduction process of Bi_2_O_3_/Co_3_O_4_ catalysts (S2–S4) became complicated with the introduction of Bi_2_O_3_. The reduction peak at 487 °C became wider and shifted towards to the lower temperature, especially on the Bi_2_O_3_/Co_3_O_4_ catalyst with the highest Bi loading amount (S4). In addition, two new peaks around 342 °C (peak I) and 402 °C (peak II) respectively ascribed to the reduction of Co^3+^ to Co^2+^, Co^2+^ to metallic Co appeared on Bi_2_O_3_/Co_3_O_4_ catalysts (S2–S4).^28^ Compared with the bulk Co_3_O_4_ (S1), both the reduction peak of Co^3+^ and that of Co^2+^ shifted to lower temperature after the deposition of Bi_2_O_3_, implying the promoted reduction of Co_3_O_4_. Moreover, the larger reduction peak I than peak II on S2–S4 samples indicated that the ratio of Co^3+^/Co^2+^ was higher on S2–S4 samples than that on the Co_3_O_4_ (S1). It revealed that the deposition of Bi_2_O_3_ on Co_3_O_4_ affected the oxidized state of cobalt in the synthesized Co_3_O_4_, more Co^3+^ were present on the supported samples (S2–S4) than the pure Co_3_O_4_.

### 3.3 X-ray photoelectron spectroscopy (XPS)

**Fig. 4:**
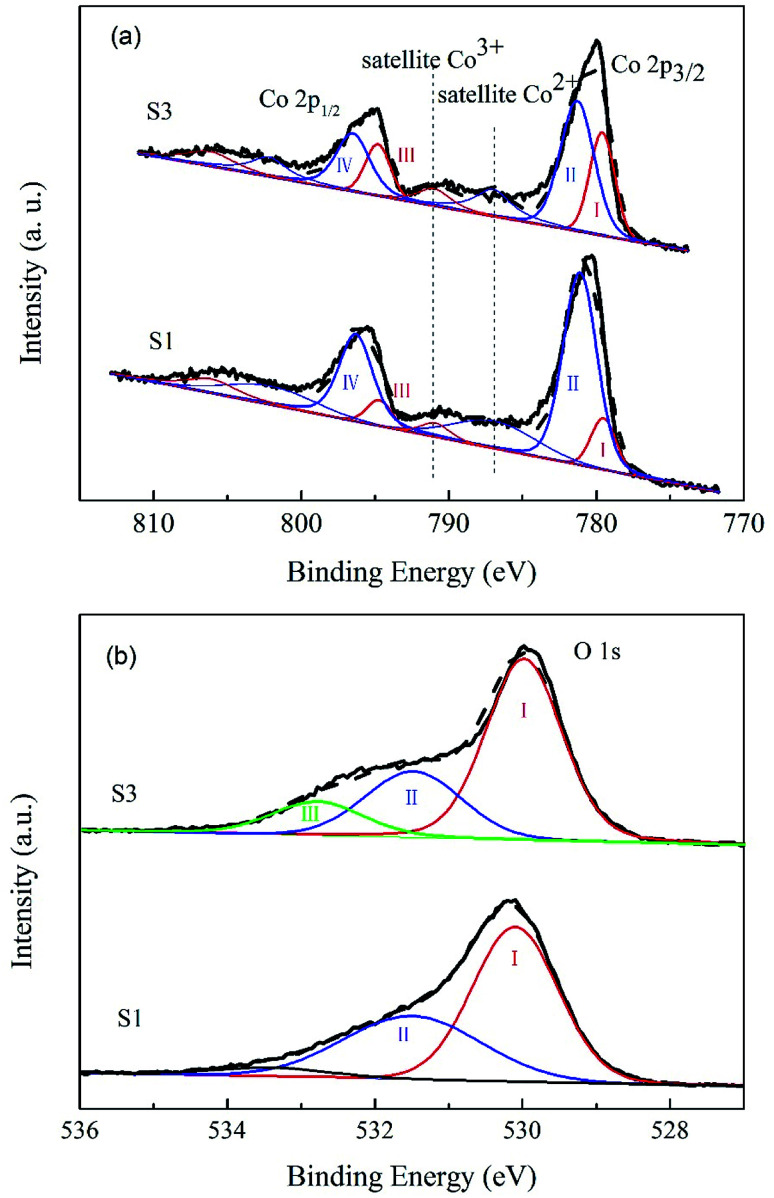
XPS study of Co_3_O_4_ (S1) and 10.0% Bi_2_O_3_/Co_3_O_4_ (S3): (a) Co 2p spectra, (b) O 1s spectra.

XPS measurements were carried out on Co_3_O_4_ and 10.0% Bi_2_O_3_/Co_3_O_4_ catalysts to examine the influence of Bi_2_O_3_ on the surface electronic state of Co_3_O_4_. The Co 2p and O 1s XPS profiles are shown in Fig. 4. In the Co 2p (Fig. 7(a)), the main peaks located at 779.6–781.3 eV and 794.8–796.5 eV are ascribed to Co 2p_1/2_ and Co 2p_3/2_ spin-orbital peaks, respectively.^29,30^ It was well-known that the spin–orbit splitting value for Co^3+^ compounds is 15.0 eV, 15.1–15.3 eV for the mixed-valence Co_3_O_4_. Here, the spin–orbit splitting values of Co 2p for CO_3_O_4_ and 10.0% Bi_2_O_3_/Co_3_O_4_ are the same, 15.2 eV, which is close to that of mixed-valence Co_3_O_4_. So the cobalt species on both S1 and S3 should be Co_3_O_4_, agreeable with the XRD and TPR results.

**Fig. 5:**
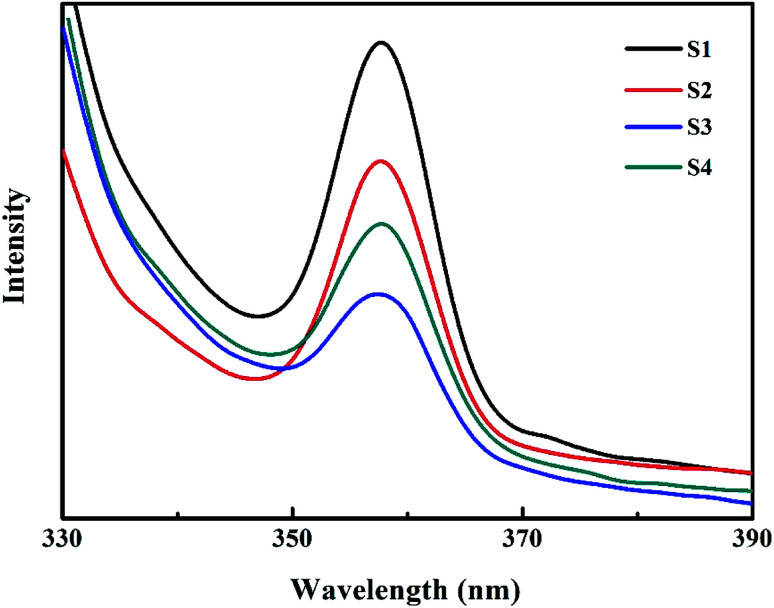
Room-temperature PL spectra of Co_3_O_4_ (S1), 5.0% Bi_2_O_3_/Co_3_O_4_ (S2), 10.0% Bi_2_O_3_/Co_3_O_4_ (S3) and 15.0% Bi_2_O_3_/Co_3_O_4_ (S4).

**Fig. 6:**
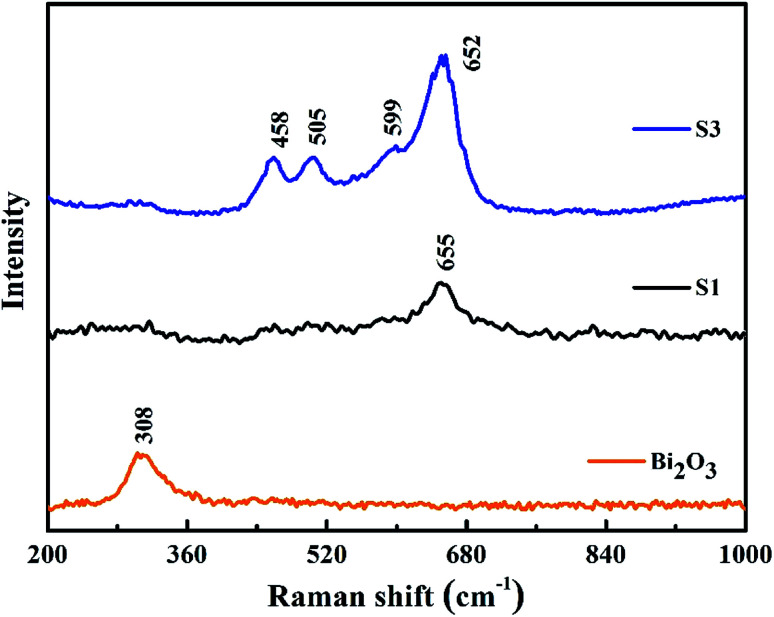
Raman spectra of Co_3_O_4_ (S1) and 10.0% Bi_2_O_3_/Co_3_O_4_ (S3).

**Fig. 7:**
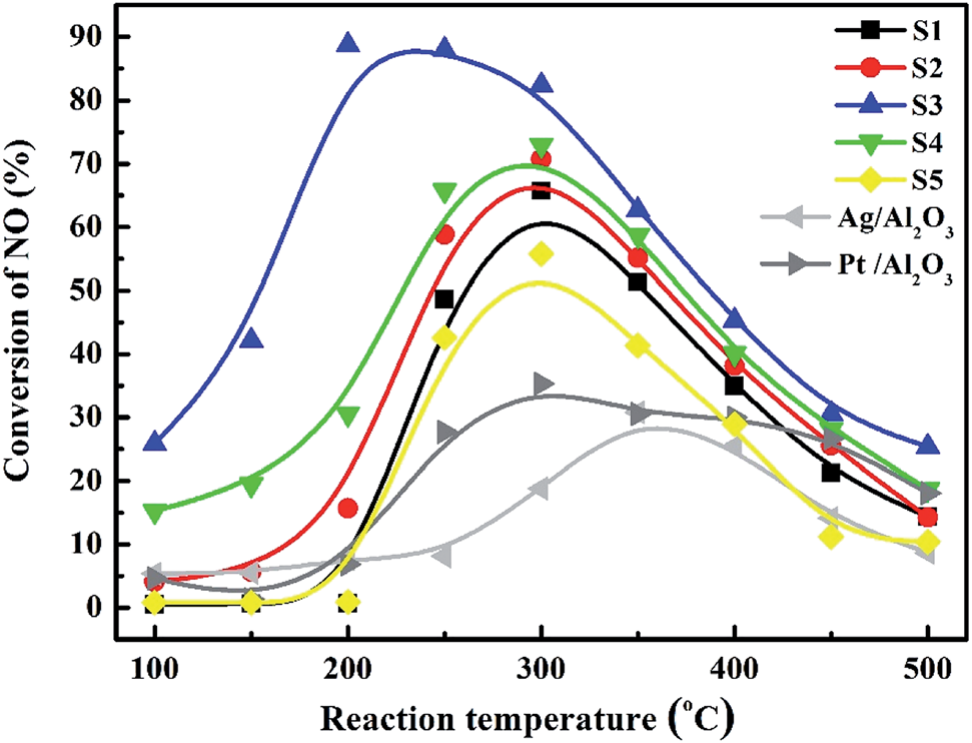
NO conversions over the catalysts: S1: Co_3_O_4_; S2: 5.0% Bi_2_O_3_/Co_3_O_4_; S3: 10.0% Bi_2_O_3_/Co_3_O_4_; S4: 15.0% Bi_2_O_3_/Co_3_O_4_; S5: the physical mixture of Bi_2_O_3_ and Co_3_O_4_.

Based on the restriction that Co 2p_3/2_ binding energies of Co^2+^ and Co^3+^ components are 781.3 eV and 779.6 eV, respectively, the spin–orbit doublet splitting is 15.2 eV with a fixed ratio of 2/1 for the 2p_3/2_-to-2p_1/2_ peak area,^29^ the Co 2p spectra of S1 and S3 can be fitted to the Co^2+^ (peak II and IV) and Co^3+^ (peak I and III) components.^30,31^ The satellite peak of Co^2+^ and that of Co^3+^ in Co_3_O_4_ were also respectively observed at 787.0 eV and 791.0 eV.^32,33^Fig. 4 shows that the peak areas of peak I and III increased with the addition of Bi_2_O_3_, implying that the surface Co^3+^ and the surface content ratio of Co^3+^/Co^2+^ increased with the addition of Bi_2_O_3_. More Co^3+^ was present on 10.0% Bi_2_O_3_/Co_3_O_4_ (S3) than the bulk Co_3_O_4_ support (S1), as consistent with the TPR results.

The O 1s XPS spectra of Co_3_O_4_ and Bi_2_O_3_/Co_3_O_4_ catalysts are shown in Fig. 4(b). For the Co_3_O_4_ sample, there are two peaks (I and II). The peak I located at ∼530.1 eV is attributed to the surface lattice of Co_3_O_4_, and the peak II at ∼531.5 eV is associated with OH^−^ groups.^34^ In the case of 10.0% Bi_2_O_3_/Co_3_O_4_, besides the peak I and II, one new peak at ∼532.8 eV appeared, which should be related to the contribution of the oxygen from Bi_2_O_3_.

### 3.4 Photoluminescence (PL) and Raman spectra

PL emission spectra originating from the recombination of free charge carriers are useful to reveal the migration, transfer and separation of photogenerated charge carriers. Fig. 5 shows photoluminescence emission spectra of different catalysts at room temperature. All samples show one luminescence peak center at about 358 nm, which can be attributed to the radiative recombination of charge carriers. The pure Co_3_O_4_ has the strongest PL emission peak. This charge recombination process of Co_3_O_4_ can be greatly inhibited by the deposition of Bi_2_O_3_ on Co_3_O_4_. 10.0% Bi_2_O_3_/Co_3_O_4_ (S3) has the lowest PL emission peak, which is associated with its structural imperfection. The structural imperfection originating from the insertion of the Bi^3+^ into the lattice of Co_3_O_4_, as evidenced by XRD, increased the number of structural defects (*e.g.*, oxygen vacancies), which could capture the electrons or holes, thus resulting in low radiative PL emission.

Fig. 6 shows the Raman spectra of 10.0% Bi_2_O_3_/Co_3_O_4_ (S3), pure Co_3_O_4_ (S1) and Bi_2_O_3_. For the pure Co_3_O_4_, the Raman peak at 655 cm^−1^ was corresponded to the symmetry of Co_3_O_4_.^35^ 10.0% Bi_2_O_3_/Co_3_O_4_ gave the similar Raman spectra with Co_3_O_4_, while the characteristic peaks of Bi_2_O_3_ could not be detected. Compared with Co_3_O_4_, the Raman peaks on 10.0% Bi_2_O_3_/Co_3_O_4_ shifted to the lower frequencies with stronger intensities, which associated with the lattice distortion or residual stress of the spinel structure. The XRD results showed that part of Bi_2_O_3_ entered the lattice of Co_3_O_4_ over 10.0% Bi_2_O_3_/Co_3_O_4_, leading to the lattice distortion and lattice defect. The highly defective structure formed on 10.0% Bi_2_O_3_/Co_3_O_4_ could accelerate the adsorption and activation of O_2_, which was suggested to be related to the better catalytic performance.

### 3.5 Catalytic performance

Fig. 7 depicted the NO conversions over the Bi_2_O_3_/Co_3_O_4_ catalysts with different Bi_2_O_3_ contents (S1–S4), the physical mixture of 10% Bi_2_O_3_ nanoparticles and Co_3_O_4_ support (S5), 4% Ag/Al_2_O_3_ and 2% Pt/Al_2_O_3_ reference catalysts within the reaction temperature range of 100–500 °C. Co_3_O_4_ and Bi_2_O_3_/Co_3_O_4_ catalysts showed higher NO reduction activity than those of Ag/Al_2_O_3_ and Pt/Al_2_O_3_, especially Bi_2_O_3_/Co_3_O_4_ catalysts. The conversion of NO over Co_3_O_4_ support (S1) firstly increased with reaction temperature, reached the maximum conversion (*ca.* 60%) at *ca.* 300 °C and then decreased at higher temperature. The NO conversion was further increased with the addition of Bi_2_O_3_ into Co_3_O_4_ with the activity order: Co_3_O_4_ (S1) < 5.0% Bi_2_O_3_/Co_3_O_4_ (S2) < 15.0% Bi_2_O_3_/Co_3_O_4_ (S4) < 10.0% Bi_2_O_3_/Co_3_O_4_ (S3). Among all catalysts tested, 10.0% Bi_2_O_3_/Co_3_O_4_ (S3) possessed the highest activity for NO conversion in the reaction temperature window, reaching *ca.* 90% NO conversion at 200 °C. NO conversion under the lower reaction temperature (100–250 °C) over S3 also reached the highest among the catalysts tested. In contrast, the mixture of 10% Bi_2_O_3_ nanoparticles and Co_3_O_4_ support (S5) showed lower activity than S1 and S3. It was indicated that the interaction between Bi_2_O_3_ and Co_3_O_4_ in S3 is not the simply physical mixture like S5. The chemical interaction between them took place in S3 and should contribute the admirable catalytic performance of S3 in the C_3_H_6_-SCR reaction.

**Fig. 8:**
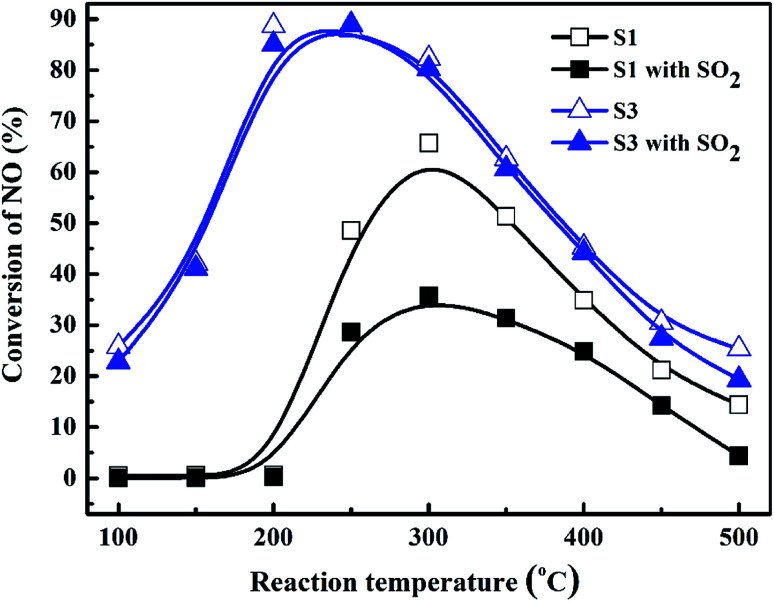
SO_2_ resistibilities of Co_3_O_4_ (S1) and 10.0% Bi_2_O_3_/Co_3_O_4_ (S3) with the reaction temperature.

SO_2_ usually exists in the diesel engine exhaust. So it is necessary to investigate the SO_2_ tolerance of the catalyst in C_3_H_6_-SCR. Fig. 8 exhibited the effects of 100 ppm SO_2_ co-fed in the reaction gas on the NO conversions over the catalysts at the different reaction temperatures. NO conversion over the S3 (10% Bi_2_O_3_/Co_3_O_4_) catalyst clearly did not change in the wide reaction window. The steady-state NO conversion reached 90.3% on S3 at 250 °C in the presence and absence of SO_2_. In contrast, NO conversion decreased from 65.8% to 35.7% at 300 °C on the Co_3_O_4_ support (S1) when 100 ppm SO_2_ was contained in the feed gas. NO conversions at other reaction temperatures also reduced in the presence of SO_2_. These results obviously suggested that 10% Bi_2_O_3_/Co_3_O_4_ exhibited good resistibility against SO_2_ that coexists with NO and C_3_H_6_ in the reaction mixture.

**Fig. 9:**
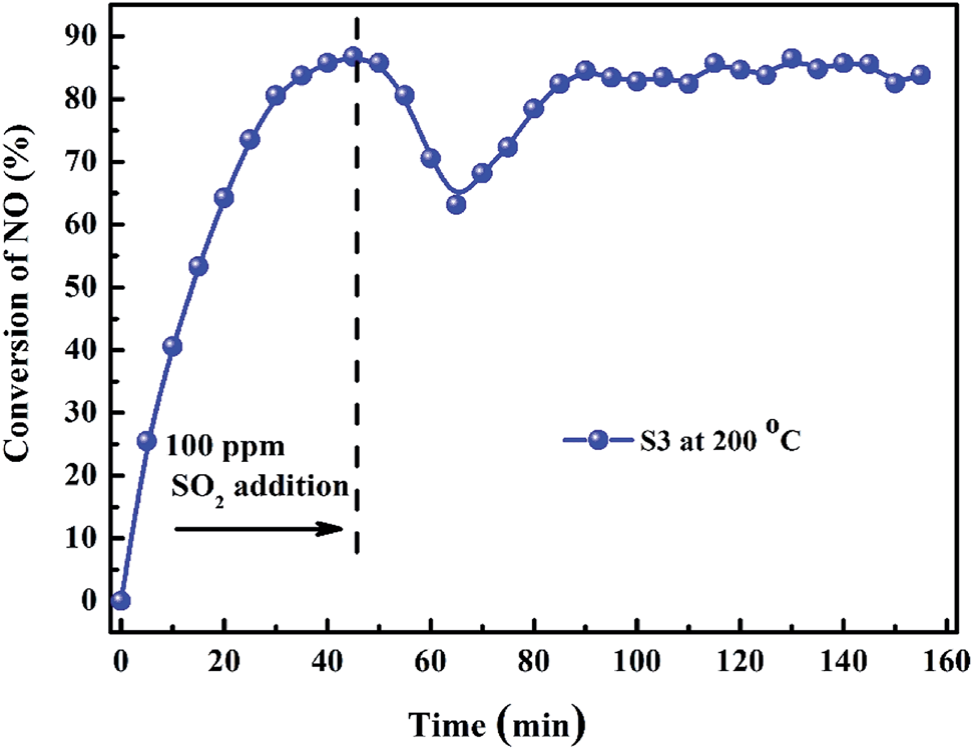
NO conversion over 10.0% Bi_2_O_3_/Co_3_O_4_ (S3) before and after the addition of SO_2_ (reaction temperature: 200 °C).

Fig. 9 shows the SO_2_ durability of 10% Bi_2_O_3_/Co_3_O_4_ catalyst with the reaction time at the optimum reaction temperature 200 °C in the C_3_H_6_-SCR of NO. When NO conversion reached to the maximum (89.3%), 100 ppm SO_2_ was added in the reaction system, NO conversion immediately decreased. It was probably due to the competitive adsorption of NO and SO_2_ on the active site. 20 min later, NO conversion reduced to 63.6%. After that, NO conversion recovered to 85.7%, and maintained at *ca.* 88% through the whole reaction period of 90 min. This result further illustrates the outstanding SO_2_ resistibility of 10% Bi_2_O_3_/Co_3_O_4_ in the long time-reaction.

## 4 Discussions

In this study, the deposition of Bi_2_O_3_ with the proper loading amount on Co_3_O_4_ nanoplates enhanced NO conversion over Co_3_O_4_, especially at low reaction temperature (<200 °C). 10.0% Bi_2_O_3_/Co_3_O_4_ catalyst also showed the strong resistibility against SO_2_ in the feed gas. XRD results showed Bi_2_O_3_ could enter the lattice of Co_3_O_4_, and promote the formation of the lattice distortion and structural defect as demonstrated by PL spectra and IR spectra. The H_2_-TPR and XPS results showed that more Co^3+^ appeared with the deposition of Bi_2_O_3_. These changes were probably related to the promotive effects of Bi_2_O_3_ in the C_3_H_6_-SCR reaction.

**Scheme 1:**
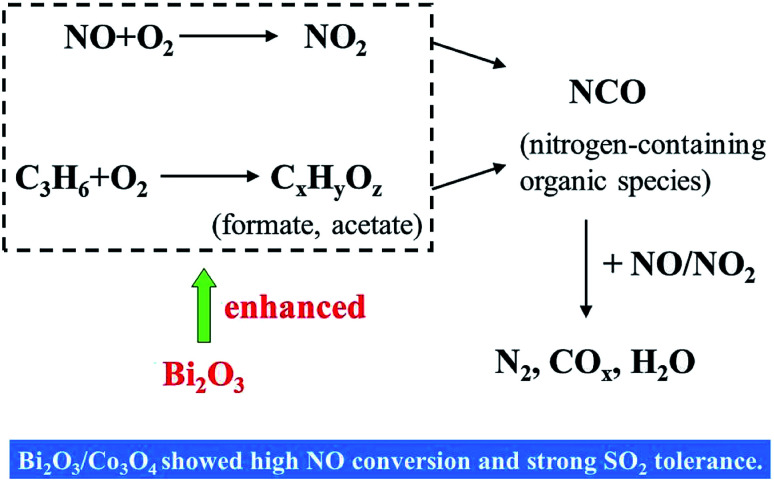
The promotive effects of Bi_2_O_3_ on NO reduction by propene over Co_3_O_4_ catalyst.

Scheme 1 illustrated the mechanistic investigations for the HC-SCR reactions in the previous literatures.^17,36,37^ According to these findings, the reactants (C_3_H_6_, NO and NO_2_) are supposed to be first adsorbed on the active sites over the catalyst surface. Subsequently, the adsorbed nitrates formed *via* NO oxidation by O_2_. C_3_H_6_ was also activated to form C_*x*_H_*y*_O_*z*_ species such as formate, acetate and so on. As these C_*x*_H_*y*_O_*z*_ species become available, nitrates subsequently reacted with them to yield nitrogen-containing organic species, such as NCO species. The final step would be the interaction of NOC intermediates with NO_*x*_ (NO, NO_*x*_), decomposing into N_2_, CO_*x*_ and H_2_O as final products. This proposed reaction process reveals the crucial role of O_2_ in the feed gas and the importance of oxidizing characteristics of the catalyst surface.

In our work, PL and IR results showed that more oxygen vacancies were produced on the Co_3_O_4_ after the doping of Bi_2_O_3_. The vacancy could accelerate the adsorption, activation and diffusion of oxygen, which was suggested to be available for the oxidation reactions involved in HC-SCR. The doped Bi_2_O_3_ also increased the Co^3+^ concentration on the surface. The richness of Co^3+^ could promote the adsorption and activation of NO and (or) C_3_H_6_. What is more is the mild oxidizing characteristics of bismuth oxide in the selective oxidation and ammoxidation of propene to acrolein and acrylonitrile. It will accelerate the formation of C_*x*_H_*y*_O_*z*_ species. In short, the addition of Bi_2_O_3_ into the Co_3_O_4_ in the present study probably influenced the oxidation process in the C_3_H_6_-SCR reaction, favored the activation of C_3_H_6_ and NO, and then enhanced the following NCO intermediate formation and its decomposition with reaction with NO_*x*_ to N_2_.

About poisoning HC-SCR catalyst with SO_2_, the previous studies reported that the suppression effect of SO_2_ on the SCR catalyst could attributed to the formation of sulphate on the catalyst.^38^ The presence of surface SO_4_^2−^ groups blocked the formation of nitrate and decreased the amount of adsorbed nitrates. Thus it hindered the transformation of NOC species and decreased the catalytic activity. In our work, the presence of 100 ppm SO_2_ in the feed gas decreased the NO conversion over Co_3_O_4_ catalyst, while the SO_2_ tolerance of Bi_2_O_3_/Co_3_O_4_ was strong. 10% Bi_2_O_3_/Co_3_O_4_ showed the good stability when SO_2_ was co-fed in the mixture gas during 90 min. This promotive role of Bi_2_O_3_ on the resistibility against SO_2_ also could be explained by the oxidizing properties of 10% Bi_2_O_3_/Co_3_O_4_. The addition of Bi_2_O_3_ into Co_3_O_4_ promoted the partial oxidation of propene activation of C_3_H_6_. The activation of C_3_H_6_ over 10% Bi_2_O_3_/Co_3_O_4_ seems more competitive in the compassion with the oxidization of SO_2_ to form surface sulfate. Subsequently, it is suggested that the addition of Bi_2_O_3_ into Co_3_O_4_ is one appropriate method for improving the SO_2_ resistance of Co_3_O_4_.

## 5 Conclusions

The optimum Bi_2_O_3_ loading on the Co_3_O_4_ nanoplates for the C_3_H_6_-SCR of NO was about 10%, giving the best catalytic activity, especially at low reaction temperature, as well as the strongest SO_2_ tolerance. The decoration of moderate Bi_2_O_3_ on Co_3_O_4_ influenced the oxidation state of Co_3_O_4_, facilitate the surface oxygen mobility and the partial oxidation of propene involved in the C_3_H_6_-SCR reaction. Therefore, the combination of Co_3_O_4_ with Bi_2_O_3_ is more active than Co_3_O_4_. The addition of 100 ppm SO_2_ to the feed hardly affected the catalytic performance of 10% Bi_2_O_3_/Co_3_O_4_.

## Conflicts of interest

There are no conflict to declare.

## Supplementary Material
